# Probabilities and Epistemic Operations in the Logics of Quantum Computation

**DOI:** 10.3390/e20110837

**Published:** 2018-10-31

**Authors:** Maria Luisa Dalla Chiara, Hector Freytes, Roberto Giuntini, Roberto Leporini, Giuseppe Sergioli

**Affiliations:** 1 Dipartimento di Lettere e Filosofia, Università di Firenze, Via Bolognese 52, I-50139 Firenze, Italy; 2 Dipartimento di Pedagogia, Psicologia, Filosofia, Università di Cagliari, Via Is Mirrionis 1, I-09123 Cagliari, Italy; 3 Dipartimento di Ingegneria Gestionale, dell’Informazione e della Produzione, Università di Bergamo, viale Marconi 5, I-24044 Dalmine (BG), Italy

**Keywords:** quantum logics, quantum probability, holistic semantics, epistemic operations

## Abstract

Quantum computation theory has inspired new forms of quantum logic, called *quantum computational logics*, where formulas are supposed to denote pieces of quantum information, while logical connectives are interpreted as special examples of quantum logical gates. The most natural semantics for these logics is a form of *holistic semantics*, where meanings behave in a contextual way. In this framework, the concept of *quantum probability* can assume different forms. We distinguish an absolute concept of probability, based on the idea of *quantum truth*, from a relative concept of probability (a form of *transition-probability*, connected with the notion of fidelity between quantum states). Quantum information has brought about some intriguing epistemic situations. A typical example is represented by teleportation-experiments. In some previous works we have studied a quantum version of the epistemic operations “to know”, “to believe”, “to understand”. In this article, we investigate another epistemic operation (which is informally used in a number of interesting quantum situations): the operation “being probabilistically informed”.

## 1. Introduction

Quantum information and quantum computation have inspired new developments of some basic concepts of the quantum theoretic formalism, which for a long time had been regarded as mysterious and potentially paradoxical. In this framework the concept of *quantum probability* has been investigated according to new perspectives, giving rise to possible applications to fields that are far apart from microphysics (cognitive and social sciences, semantics of natural languages and of the languages of art, see, for instance, [1,2,3]).

As is well known, the basic idea of quantum computation theory is that information can be stored and transmitted by quantum physical objects. Accordingly, pieces of quantum information can be identified with states of some special quantum systems that are storing the information in question. In the simplest case a piece of quantum information corresponds to a pure state of a single particle: a qubit (or qubit-state), the quantum counterpart of the classical concept of bit. Mathematically a qubit can be represented as a quantum superposition (living in the two-dimensional Hilbert space $C^{2}$), whose form is

$$|\psi \rangle =c_{0}|0\rangle +c_{1}|1\rangle ,$$

where |0〉 and |1〉 are the two elements of the canonical orthonormal basis of the space, representing in this framework the two classical truth-values 1 (Truth) and 0 (Falsity). From an intuitive point of view, any qubit

$$c_{0}|0\rangle +c_{1}|1\rangle$$

can be regarded as un uncertain information that might be true with probability ${\left|c_{1}\right|}^{2}$ and might be false with probability ${\left|c_{0}\right|}^{2}$. More generally, a piece of quantum information corresponds to a complex knowledge that can be mathematically represented as a pure or mixed state of a composite quantum system: a density operator ρ of a finite-dimensional Hilbert space whose standard form is

$$H^{\left(n\right)}=\underset{n-times}{\underset{︸}{C^{2}⊗\ldots ⊗C^{2}}}\;(\mathrm{the}\;n-\mathrm{fold}\;\mathrm{tensor}\;\mathrm{product}\;\mathrm{of}\;C^{2}).$$

Quantum information is processed by (quantum logical) gates: special examples of unitary quantum operations that transform pure and mixed states in a reversible way. Any finite sequence of gates (defined on a space $H^{\left(n\right)}$) gives rise to a quantum circuit: when applied to a given input $\rho_{in}$, the circuit under consideration transforms $\rho_{in}$ into an output $\rho_{out}$. This represents a mathematical description for a physical process that might be performed by a quantum computer.

The theory of quantum circuits has inspired a natural logical abstraction, giving rise to the development of new forms of quantum logic that have been termed *quantum computational logics*. In these logics, formulas are supposed to denote pieces of quantum information, while logical connectives are interpreted as special examples of gates. Consequently, all formulas of quantum computational languages turn out to have a typical dynamic character, representing possible computation-actions. The most natural semantics for *quantum computational logics* is a form of *holistic semantics*, where *quantum entanglement* (often described as “mysterious”) can be used as a powerful logical resource. Against the classical compositionality-principle, meanings of well-formed expressions of a quantum computational language behave in a holistic and contextual way: the meaning of a global expression determines the meanings of its well-formed parts, and not the other way around. Furthermore, meanings are generally context-dependent: under one and the same interpretation of the language an expression may receive different meanings in different contexts (as, in fact, happens in our current use of natural languages and in many forms of informal reasoning).

An important “character” of the quantum computational semantics is the concept of *quantum probability*, which can assume different forms. A basic notion of *quantum probability* (which plays an important logical role) is connected with the idea of truth. In any quantum computational space $H^{\left(n\right)}$ the concept of truth can be naturally represented as a special projection operator, indicated by $P_{1}^{\left(n\right)}$. For instance, in the case of the space $H^{\left(1\right)}=C^{2}$ the truth-concept $P_{1}^{\left(1\right)}$ is identified with the projection $P_{|1\rangle }$ that projects over the closed subspace determined by the bit |1〉 (corresponding to the classical truth-value 1). In this way, truth is dealt with as a special example of a mathematical representative for a possible physical event. Accordingly, one can naturally apply the basic probabilistic rule of quantum theory, based on the concept of Born-probability. For any qubit $|\psi \rangle =c_{0}|0\rangle +c_{1}|1\rangle$, the probability that the quantum information |ψ〉 satisfies the *truth* can be defined as follows:

$$p_{1}(|\psi \rangle ):=∥P_{|1\rangle }{|\psi \rangle ∥}^{2}={\left|c_{1}\right|}^{2}=\mathrm{tr}\left(P_{|\psi \rangle }P_{1}^{\left(1\right)}\right),$$

where tr is the *trace functional* and $∥P_{|1\rangle }|\psi \rangle ∥$ is the length of the vector obtained by projecting |ψ〉 over the closed subspace determined by |1〉. In the next Section we will see how the probability-function $p_{1}$ can be generalized to all pieces of quantum information, living in any space $H^{\left(n\right)}$.

Interestingly enough, the contextual properties of the holistic quantum computational semantics allow us to understand and to justify (at least to a certain extent) some strange uses of the concept of probability that sometimes occur in the framework of intuitive ways of reasoning (for a general discussion of this problem see, for instance, [1]). For instance, assigning to a conjunction α∧β a probability-value greater than the probabilities of both members (α,β) is not necessarily “irrational” or “antiscientific”. For, it might happen that the meanings of the three sentences α∧β, α, β refer, in fact, to different contexts.

Once fixed the truth-concept $P_{1}^{\left(n\right)}$ (for any space $H^{\left(n\right)}$), the probability-function $p_{1}$ represents a kind of absolute concept of probability: any piece of quantum information has a well-determined probability-value of being true. It is interesting to investigate another concept of probability that represents a form of relative probability. Suppose that the pure state

$$|\psi \rangle =\sum_{i}c_{i}|\varphi_{i}\rangle$$

(where every $|\varphi_{i}\rangle$ is an element of the canonical orthonormal basis of the space $H^{\left(n\right)}$) represents the information of an epistemic agent at a given time (say, at the beginning of an experiment or of a computation). According to the quantum theoretic formalism, the information |ψ〉 allows us to assign probability-values to other pieces of information, that may represent possible outcomes of a measurement or of a computation. For instance, the probability that the information |ψ〉 assigns to the outcome $|\varphi_{i}\rangle$ is ${\left|c_{i}\right|}^{2}$. Thus, whenever we have the information |ψ〉 we might have the information $|\varphi_{i}\rangle$ with probability ${\left|c_{i}\right|}^{2}$. Accordingly, we can write:

$$p_{|\psi \rangle }\left(|\varphi_{i}\rangle \right)={\left|c_{i}\right|}^{2}.$$

We will see how this concept of relative probability (which can be generalized to mixed states) is strongly connected with the concept of fidelity between quantum states.

Quantum information has brought about some intriguing epistemic situations. A typical example is represented by teleportation-experiments, where an agent (say, Alice) transmits qubit-states to a far agent (say, Bob), by using some special quantum non-locality phenomena, which may appear *prima facie* strange and mysterious. These puzzling (non-classical) situations have inspired new ideas in the field of epistemic logics. See, for instance, [4].

As is well known, many standard approaches to epistemic logics have been developed in the framework of a possible world semantics, where the basic epistemic operators (“to know”, “to believe”) are dealt with as special examples of modal operators. Is it possible to represent epistemic operators as particular examples of quantum operations in the framework of a quantum computational semantics? This question admits a positive answer. In some previous works we have studied a quantum version of the epistemic operators “to know”, “to believe”, “to understand”, whose semantic properties depend on the notion of *quantum truth* and on the probability-function $p_{1}$. We have seen, in particular, how quantum knowledge generally gives rise to forms of “reversibility-breaking” that can be compared with what happens in the case of quantum measurements. In this article we will investigate another epistemic concept: the operation “being probabilistically informed”, which plays a significant role in a number of interesting quantum situations (for instance, in the case of quantum teleportation-experiments).

## 2. Pieces of Quantum Information and Quantum Probabilities

We will first recall the basic “mathematical characters” that play an important role in quantum computation. The “mathematical stage” where any piece of quantum information is usually supposed to live is an *n*-fold tensor product of the Hilbert space $C^{2}$:

$$H^{\left(n\right)}=\underset{n-times}{\underset{︸}{C^{2}⊗\ldots ⊗C^{2}}}.$$

Any piece of quantum information is a special mathematical object that lives in a particular quantum computational space $H^{\left(n\right)}$, representing a possible state of a physical system that is storing the information in question. For some applications it may be useful to take, as a basic quantum computational space, a many-dimensional space $C^{d}$ (with d>2). In this way, qubits are generalized to qudits. See, for instance, [5]. Important examples are represented by quregisters, qubits, registers, bits and mixtures of quregisters. In the quantum computation-literature the terms “qubit” (or “quantum bit”) and “quregister” (or “quantum register”) are sometimes used in an ambiguous way. In many cases the expression “qubit” refers to a quantum system (say, an electron or a photon) whose possible pure states live in the space $C^{2}$. In some other cases, instead, “qubit” simply means a possible pure state of such a system. A similar ambiguity regards the term “quregister”. In this article, we will always use the terms “qubit” and “quregister” in the sense of possible pure states of quantum systems that can store pieces of quantum information.

**Definition** **1** (Quregisters and registers)**.**

- *A* quregister (*or* quregister-state) *is a unit vector of a space* $H^{\left(n\right)}$.
- *A* qubit (*or* qubit-state) *is a quregister of the space* $C^{2}$.
- *A* register (*or* register-state) *is an element*
  $$|x_{1},\ldots ,x_{n}\rangle =|x_{1}\rangle ⊗\ldots ⊗|x_{n}\rangle$$
  *of the canonical orthonormal basis of the space* $H^{\left(n\right)}$ (*where* $x_{i}\in \left\{0,1\right\}$).
- *A* bit *is a register of the space* $C^{2}$.

Any quregister |ψ〉 of the space $H^{\left(n\right)}$ can be represented as a quantum superposition of registers that belong to the canonical basis of the space:

$$|\psi \rangle =\sum_{i}c_{i}|x_{i_{1}},\ldots ,x_{i_{n}}\rangle ,$$

where $c_{i}$ are complex numbers (called amplitudes) such that $\sum_{i}{\left|c_{i}\right|}^{2}=1$.

Quregisters are pure states representing maximal pieces of information (about the quantum systems under investigation) that cannot be consistently extended to a richer knowledge. More generally, a piece of quantum information may correspond to a non-maximal knowledge: a mixed state (or mixture of quregisters), that is mathematically represented as a density operator ρ of a space $H^{\left(n\right)}$. Of course, any quregister |ψ〉 corresponds to a special case of a density operator: the projection $P_{|\psi \rangle }$ that projects over the closed subspace determined by |ψ〉. We will indicate by $D(H^{\left(n\right)})$ the set of all density operators of the space $H^{\left(n\right)}$, while $D={⋃}_{n}\left\{D(H^{\left(n\right)})\right\}$ will represent the set of all possible pieces of quantum information.

A piece of quantum information is generally stored by a composite system *S* consisting of some subsystems $S_{1},\ldots S_{r}$, where each part $S_{i}$ may be, in turn, a composite system. According to the quantum theoretic formalism any possible (pure or mixed) state ρ of *S* determines the state $\rho_{i}$ of each subsystem $S_{i}$; this state is called the reduced state of ρ with respect to the *i*-th subsystem. Of course, some composite systems *S* might be decomposed into parts in different ways; thus, the mathematical formalism shall take into account all possible decomposition-choices.

Consider a Hilbert space $H^{\left(n\right)}$ that can be decomposed as

$$H^{\left(n\right)}=H^{\left(m_{1}\right)}⊗\ldots ⊗H^{\left(m_{r}\right)},$$

where $m_{1}+\ldots +m_{r}=n$. Accordingly, any density operator ρ of $H^{\left(n\right)}$ can be regarded as a possible state of a composite system

$$S=S_{1}+\ldots +S_{r},$$

where $H^{\left(m_{i}\right)}$ is the Hilbert space associated to the subsystem $S_{i}$. Consider now a particular subsystem of *S*:

$$S_{i_{1}}+\ldots +S_{i_{k}}$$

(with $1\leq i_{1},\ldots ,i_{k}\leq r$). We will indicate by

$$Red_{\left[m_{1},\ldots ,m_{r}\right]}^{\left(i_{1},\ldots ,i_{k}\right)}$$

the reduced state of ρ with respect to the subsystem $S_{i_{1}}+\ldots +S_{i_{k}}$ and with respect to the decomposition $H^{\left(n\right)}=H^{\left(m_{1}\right)}⊗\ldots ⊗H^{\left(m_{r}\right)}$. By simplicity we will omit the subscript $\left[m_{1},\ldots ,m_{r}\right]$ in the case where the decomposition of $H^{\left(n\right)}$ is obvious.

The mathematical representation of composite systems (via tensor products) has brought about some deep changes in the relationships between parts and whole in the quantum world. As is well known, in perfect harmony with the semantics of classical logic, classical physical systems satisfy a physical compositionality-principle: the states of the subsystems of a given system *S* determine the state of *S* and vice versa (from the parts to the whole and from the whole to the parts). In quantum theory the compositionality-principle is strongly violated: the state of a composite system determines the state of all its parts, but generally not the other way around. The mysterious *quantum entanglement* (which has for a long time regarded as potentially paradoxical) is connected with the failure of the compositionality-principle.

What exactly is entanglement? For the sake of simplicity, in this article we will only consider the case of entangled pure states.

**Definition** **2** (Entangled pure state)**.** *Consider a composite quantum system*

$$S=S_{1}+\ldots +S_{r},$$

*and let* $H_{S},\;\;\;H_{S_{1}},\ldots ,H_{S_{r}}$ *be the Hilbert spaces associated to the systems* $S,S_{1},\ldots ,S_{r}$ *(respectively). A pure state* |ψ〉 *of S is called* entangled *iff* |ψ〉 *cannot be represented as a* factorized state

$$|\psi_{1}\rangle ⊗\ldots ⊗|\psi_{r}\rangle ,$$

*where* $|\psi_{1}\rangle ,\ldots |\psi_{r}\rangle$ *belong to the spaces* $H_{S_{1}},\ldots H_{S_{1}}$*, respectively.*

Important examples of entangled pure states are the so called Bell-states, that live in the space $H^{\left(2\right)}=C^{2}⊗C^{2}$.

**Example** **1.** *A typical Bell-state is the following:*

$$|\psi \rangle =\frac{1}{\sqrt{2}}|0,0\rangle +\frac{1}{\sqrt{2}}|1,1\rangle .$$

*Apparently,* |ψ〉 *describes the state of a two-particle system (* $S=S_{1}+S_{2}$ *), assigning probability-value* $\frac{1}{2}$ *to the two following possibilities:*

- *both subsystems are in the state |0〉;*
- *both subsystems are in the state |1〉.*

*One can show that the two reduced states of* |ψ〉 *(with respect to the first subsystem* $S_{1}$ *and with respect to the second subsystem* $S_{2}$ *) are one and the same mixed state:*

$$Red_{\left[1,1\right]}^{\left(1\right)}(|\psi \rangle )=Red_{\left[1,1\right]}^{\left(2\right)}(|\psi \rangle )=\frac{1}{2}I^{\left(1\right)}$$

*(where* $I^{\left(1\right)}$ *is the identity operator of the space* $H^{\left(1\right)}=C^{2}$ *).*

*Since* |ψ〉 *is a pure state, while* $\frac{1}{2}I^{\left(1\right)}$ *is a proper mixture, we obtain:*

$$P_{|\psi \rangle }\neq Red_{\left[1,1\right]}^{\left(1\right)}(|\psi \rangle )⊗Red_{\left[1,1\right]}^{\left(2\right)}(|\psi \rangle ).$$

*Thus, the states of the two parts of S turn out to be* indistinguishable and entangled *in the context* |ψ〉.

Let us now turn to the concept of *quantum probability*, a basic “character” of the quantum theoretic formalism that can assume different forms. We will first consider the concept of truth-probability that is naturally connected with the idea of quantum truth. In Section 4 we will see how this concept will play an important role in the semantics of *quantum computational logics*. As noticed in the Introduction, in any space $H^{\left(n\right)}$ the concept of truth can be identified with a special projection operator indicated by $P_{1}^{\left(n\right)}$. In this way, truth is dealt with as a mathematical representative of a possible physical event.

In order to define the truth-concept $P_{1}^{\left(n\right)}$ (of $H^{\left(n\right)}$), we will first distinguish the true registers from the false registers of the space.

**Definition** **3** (True and false registers**).** *Let* $|x_{1},\ldots ,x_{n}\rangle$ *be a register of $H^{\left(n\right)}$.*

- $|x_{1},\ldots ,x_{n}\rangle$*is called* true *iff its last bit* $|x_{n}\rangle$ *is |1〉;*
- $|x_{1},\ldots ,x_{n}\rangle$*is called* false *iff its last bit* $|x_{n}\rangle$ *is |0〉.*

Thus, the *truth-value* of a register is determined by its last bit. As we will see in the next Section, this choice turns out to be natural and useful in the theory of some important quantum logical gates, where the last bit of an input-register represents the *target* that is transformed by the gate in question into the final truth-value of the output.

On this basis one can now define the concepts of truth and falsity of a space $H^{\left(n\right)}$.

**Definition** **4** (Truth and falsity)**.**

- *The* truth *of the space* $H^{\left(n\right)}$ *is the projection* $P_{1}^{\left(n\right)}$ *that projects over the closed subspace spanned by the set of all true registers.*
- *The* falsity *of the space* $H^{\left(n\right)}$ *is the projection* $P_{0}^{\left(n\right)}$ *that projects over the closed subspace spanned by the set of all false registers.*

As a particular case, we obtain that the *truth* $P_{1}^{\left(1\right)}$ of the space $C^{2}$ is the projection $P_{|1\rangle }$ (which projects over the closed subspace determined by the bit |1〉).

Now, we can naturally apply the basic probabilistic rule of quantum theory: the Born-rule, which determines for any state ρ and for any physical event represented by a projection operator *P* (of a Hilbert space H), the probability that a quantum system in state ρ verifies the event represented by *P*. According to this rule we have:

$$Prob_{\rho }\left(P\right)=\mathrm{tr}\left(\rho P\right)$$

(where $Prob_{\rho }\left(P\right)$ represents the probability that the state ρ assigns to the event *P*).

By applying the Born-rule to the particular case of the truth-concept $P_{1}^{\left(n\right)}$ we obtain for any state ρ of $H^{\left(n\right)}$:

$$Prob_{\rho }\left(P_{1}^{\left(n\right)}\right)=\mathrm{tr}\left(\rho P_{1}^{\left(n\right)}\right).$$

From an intuitive point of view, $Prob_{\rho }\left(P_{1}^{\left(n\right)}\right)$ represents the probability that the quantum information ρ is *true*. Since $P_{1}^{\left(n\right)}$ is a constant projection operator (in the space $H^{\left(n\right)}$) we can briefly write $p_{1}\left(\rho \right)$, instead of $Prob_{\rho }\left(P_{1}^{\left(n\right)}\right)$. Once chosen the truth-concept $P_{1}^{\left(n\right)}$ (in any space $H^{\left(n\right)}$), every piece of quantum information turns out to have a well-determined probability-value of being *true*. An important property of the function $p_{1}$ is asserted by the following Lemma.

**Lemma** **1.** *For any $\rho \in D(H^{\left(n\right)})$,*

$$p_{1}\left(\rho \right)=\mathrm{tr}\left(Red_{\left[nȒ1,1\right]}^{\left(2\right)}\left(\rho \right)P_{1}^{\left(1\right)}\right).$$

As observed in the Introduction, it is interesting to consider also a different notion of quantum probability, that represents a kind of relative probability. Let us first refer to the case of quregisters (living in a given space $H^{\left(n\right)}$) and suppose that $|\psi \rangle =\sum_{i}c_{i}|x_{i_{1}},\ldots ,x_{i_{n}}\rangle$ represents the information that an epistemic agent has at a given time. The quregister |ψ〉 allows us to assign probability-values to all registers $|x_{i_{1}},\ldots ,x_{i_{n}}\rangle$ (which might represent possible outcomes of a measurement or of a computation). For any $|x_{i_{1}},\ldots ,x_{i_{n}}\rangle$, the probability that |ψ〉 assigns to the outcome $|x_{i_{1}},\ldots ,x_{i_{n}}\rangle$ is $|c_{i}{|}^{2}$. Apparently, one is dealing with a kind of *relative probability*: when we have the information |ψ〉, we might have the information $|x_{i_{1}},\ldots ,x_{i_{n}}\rangle$ with probability $|c_{i}{|}^{2}$.

In the general case, this idea of relative probability turns out to be strongly connected with the notion of fidelity: a concept (introduced by Uhlmann and by Jozsa), that represents a generalization of the notion of of transition-probability for pure states (see [6,7]).

**Definition** **5** (Fidelity)**.** *Let* |ψ〉 *and* |φ〉 *be two quregisters of* $H^{\left(n\right)}$. *The* fidelity *between* |ψ〉 *and* |φ〉 *is defined as follows:*

$$F(|\psi \rangle ,|\varphi \rangle ):={\left|\langle \psi |\varphi \rangle \right|}^{2}$$

*(where* 〈ψ|φ〉 *is the inner product of* |ψ〉 *and* |φ〉*).*

The following theorem sums up some basic properties of the notion of fidelity.

**Theorem** **1.**

*1.*  F(|ψ〉,|φ〉)∈[0,1].
*2.*  F(|ψ〉,|φ〉)=F(|φ〉,|ψ〉).
*3.*  $F(|\psi \rangle ,|\varphi \rangle )=∥P_{|\psi \rangle }|\varphi \rangle {{∥}^{2}=∥P_{|\varphi \rangle }|\psi \rangle ∥}^{2}$.
*4.*  $F(|\psi \rangle ,|\varphi \rangle )=\mathrm{tr}\left(P_{|\psi \rangle }P_{|\varphi \rangle }\right)=\mathrm{tr}\left(P_{|\varphi \rangle }P_{|\psi \rangle }\right)$.
*5.*  F(|ψ〉,|φ〉)=1*iff*$P_{|\psi \rangle }=P_{|\varphi \rangle }$.
*6.*  F(|ψ〉,|φ〉)=0*iff*|ψ〉⊥|φ〉.

From an intuitive point of view one can say that the real number F(|ψ〉,|φ〉) measures “how close” are the two pure states |ψ〉 and |φ〉 in the Hilbert space under consideration.

Since $F(|\psi \rangle ,|\varphi \rangle )=\mathrm{tr}\left(P_{|\psi \rangle }P_{|\varphi \rangle }\right)=\mathrm{tr}\left(P_{|\varphi \rangle }P_{|\psi \rangle }\right)$, recalling the Born-rule, the number F(|ψ〉,|φ〉) can be naturally interpreted as a relative probability: when we have the information |ψ〉, we might have the information |φ〉 with probability F(|ψ〉,|φ〉), or vice versa. Accordingly we can write:

$$p_{|\psi \rangle }(|\varphi \rangle )=F(|\psi \rangle ,|\varphi \rangle )=F(|\varphi \rangle ,|\psi \rangle )=p_{|\varphi \rangle }(|\psi \rangle ),$$

stressing that F(|ψ〉,|φ〉) represents the probability of the information |φ〉 under the condition |ψ〉, or vice versa. In this sense one can say that the notion of fidelity represents a special concept of conditional probability that, unlike the general case, does satisfy the symmetry-property. For a general discussion of conditional probabilities in quantum theory see [8].

The concept of fidelity (which represents a form of transition-probability in the case of pure states) can be generalized to density operators.

**Definition** **6** (Fidelity for density operators)**.** *Let ρ and σ be two density operators of $H^{\left(n\right)}$.*

$$F\left(\rho ,\sigma \right):=\mathrm{tr}{\left(\sqrt{\sqrt{\rho }\sigma \sqrt{\rho }}\right)}^{2}.$$

Interestingly enough, Jozsa has proved that this notion of fidelity between density operators coincides with the notion of transition-probability between density operators (via purification of mixtures), investigated by Uhlmann (see [7]).

The concept of fidelity for density operators represents a good generalization of the concept of fidelity for pure states, as stated by the following Lemma.

**Lemma** **2.** $$F\left(P_{|\psi \rangle },P_{|\varphi \rangle }\right)=\;\;{\left|\langle \psi |\varphi \rangle \right|}^{2}.$$

The following Theorem sums up the basic properties of the concept of fidelity for density operators.

**Theorem** **2.**

*1.*  F(ρ,σ)∈[0,1].
*2.*  F(ρ,σ)=1iffρ=σ.
*3.*  F(ρ,σ)=F(σ,ρ).
*4.*  *$F\left(U\rho U^{†},U\sigma U^{†}\right)=F\left(\rho ,\sigma \right),$ for any unitary operator U of $H^{\left(n\right)}$ (where $U^{†}$ is the adjoint of U). Thus, fidelity is preserved by unitary operators.*
*5.*  F(ρ,σ)=tr(ρσ) *, if either ρ or σ is a pure state.*

On this basis, it seems reasonable to assume that the number F(ρ,σ) represents a form of relative probability for quantum states (which may be either pure or mixed). Accordingly, we will write (like in the case of pure states):

$$p_{\rho }\left(\sigma \right)=F\left(\rho ,\sigma \right).$$

Is it possible to define fidelity for density operators that belong to different spaces? Suppose that $\rho \in D(H^{\left(n\right)})$, $\sigma \in D(H^{\left(m\right)})$ and n>m. The intuitive idea is that ρ and σ can be *compared* in the framework of the smaller space $H^{\left(m\right)}$, by referring to a special reduced state of ρ that represents a part of ρ living in $H^{\left(m\right)}$. The choice of comparing pieces of information that live in different spaces in the framework of the smaller space seems to be quite natural. In fact, both in the case of human and of artificial intelligence it often happens that agents endowed with a richer knowledge communicate with less informed agents by “reducing” their information-level to the level of the “more ignorant” agents. Accordingly, the concept of generalized fidelity can be defined as follows.

**Definition** **7** (Generalized fidelity)**.** *Let* $\rho \in D(H^{\left(n\right)})$ *and* $\sigma \in D(H^{\left(m\right)})$.

$F_{g}\left(\rho ,\sigma \right):=\left\{\begin{array}{ccc} F(\rho ,\sigma ),\;\;if\;\;n=m; & & \\ F(Red_{\left[n-m,m\right]}^{\left(2\right)}\left(\rho \right),\sigma ), & if & n>m;\\ F(\rho ,Red_{\left[m-n,n\right]}^{\left(2\right)}\left(\sigma \right)), & if & n<m. \end{array}\right.$

The map $F_{g}$ is clearly symmetric.

Thus, we can put:

$$p_{\rho }\left(\sigma \right):=F_{g}\left(\rho ,\sigma \right).$$

We obtain:

$$p_{\rho }\left(\sigma \right):=F_{g}\left(\rho ,\sigma \right)=F_{g}\left(\sigma ,\rho \right)=p_{\sigma }\left(\rho \right).$$

In this way, for any choice of ρ, the probability-function $p_{\rho }$ turns out to be defined on the set D of all possible pieces of quantum information.

It is interesting to compare the probability functions $p_{1}$ and $p_{\rho }$. As we have seen, once chosen the truth-concept in any space $H^{\left(n\right)}$, the function $p_{1}$ represents a kind of absolute concept of probability: every piece of quantum information ρ (living in whatever space) has a well-determined probability-value of being true. The probability-function $p_{\rho }$ represents, instead, a relative concept that depends on the choice of ρ. From an intuitive point of view, ρ can be regarded as an information (available for a given epistemic agent) which describes a situation that is essentially characterized by some uncertain and vague features. On the basis of his/her information our agent is able to valuate probabilistically other possible alternative situations, using the $p_{\rho }$-function.

Interestingly enough, the truth-probability function $p_{1}$ can be represented as a special case of the relative probability $p_{\rho }$.

**Theorem** **3.** *For any $\rho \in D(H^{\left(n\right)})$,*

$$p_{1}\left(\rho \right)=p_{\rho }\left(P_{1}^{\left(1\right)}\right).$$

**Proof.**  Let $\rho \in D(H^{\left(n\right)})$. By definition of $p_{1}$ we have:

$$p_{1}\left(\rho \right)=\mathrm{tr}\left(\rho P_{1}^{\left(n\right)}\right).$$

Suppose that n=1. Then,

$$p_{1}\left(\rho \right)=\mathrm{tr}\left(\rho P_{1}^{\left(1\right)}\right)=F_{g}\left(\rho ,P_{1}^{\left(1\right)}\right)=p_{\rho }\left(P_{1}^{\left(1\right)}\right).$$

Suppose that n>1. Then, by Lemma 1,

$$p_{1}\left(\rho \right)=\mathrm{tr}\left(Red_{\left[n-1,1\right]}^{\left(2\right)}\left(\rho \right)P_{1}^{\left(1\right)}\right)=F_{g}\left(\rho ,P_{1}^{\left(1\right)}\right)=p_{\rho }\left(P_{1}^{\left(1\right)}\right).$$

 □

Both the truth-probability function $p_{1}$ and the relative probability-function $p_{\rho }$ determine a preorder relation on the set D of all possible pieces of quantum information.

**Definition** **8** (The truth-preorder)**.** *For any* $\sigma_{1},\sigma_{2}\in D$,

$$\sigma_{1}⪯\sigma_{2}\;\;\;\;iff\;\;\;\;p_{1}\left(\sigma_{1}\right)\leq p_{1}\left(\sigma_{2}\right).$$

**Definition** **9** (The relative preorder)**.** *Consider a density operator ρ. For any $\sigma_{1},\sigma_{2}\in D$,*

$$\sigma_{1}{⪯}_{\rho }\sigma_{2}\;\;\;\;iff\;\;\;\;p_{\rho }\left(\sigma_{1}\right)\leq p_{\rho }\left(\sigma_{2}\right).$$

One can easily check that both relations ⪯ and ${⪯}_{\rho }$ are reflexive, transitive and generally non-antisymmetric.

## 3. Quantum Logical Circuits

The basic idea of the theory of quantum computers is that computations can be performed by some quantum objects that evolve in time. Recalling that (according to Schrödinger’s equation) the time-evolution of quantum systems is mathematically described by unitary operators, it is natural to assume that quantum information is processed by *quantum logical gates* (briefly, gates): special examples of unitary operators that transform (in a reversible way) the pure states of the quantum systems that store the information in question. Any gate $G^{\left(n\right)}$ (defined on the space $H^{\left(n\right)}$) can be canonically extended to a unitary operation ${}^{D}G^{\left(n\right)}$ (defined on the set $D(H^{\left(n\right)})$ of all density operators of $H^{\left(n\right)}$) according to the rule:

$$\forall \rho \in D\left(H^{\left(n\right)}\right):\;{\;}^{D}G^{\left(n\right)}\rho =G^{\left(n\right)}\rho \;G^{\left(n\right){\;}^{†}}$$

(where $G^{\left(n\right){\;}^{†}}$ is the adjoint of $G^{\left(n\right)}$). For the sake of simplicity, we will call gate either a unitary operator $G^{\left(n\right)}$ or the corresponding unitary operation ${}^{D}G^{\left(n\right)}$.

We will now recall the definitions of some basic gates that play an important role both from the computational and from the logical point of view. We will first consider some gates, called “semiclassical”, that cannot “create” superpositions from register-inputs. Two important examples are represented by the negation-gate and by the Toffoli-gate.

**Definition** **10** (The negation-gate on the space $H^{\left(1\right)}$)**.** *The* negation-gate *on* $H^{\left(1\right)}$ *is the linear operator* ${\mathrm{NOT}}^{\left(1\right)}$ *that satisfies the following condition for every element* |x〉 *of the canonical basis:*

$${\mathrm{NOT}}^{\left(1\right)}|x\rangle :=|1-x\rangle .$$

The operator ${\mathrm{NOT}}^{\left(1\right)}$ represents a natural quantum generalization of the classical negation. We have:

$${\mathrm{NOT}}^{\left(1\right)}|0\rangle =|1\rangle ;\;\;\;{\mathrm{NOT}}^{\left(1\right)}|1\rangle =|0\rangle .$$

The negation-gate can be naturally generalized to higher-dimensional spaces. For any $H^{\left(n\right)}$ (with n>1), the operator ${\mathrm{NOT}}^{\left(n\right)}$ is defined for every element $|x_{1},\ldots ,x_{n}\rangle$ of the canonical basis as follows:

$${\mathrm{NOT}}^{\left(n\right)}|x_{1},\ldots ,x_{n}\rangle :=|x_{1},\ldots ,x_{n-1}\rangle \;⊗\;{\mathrm{NOT}}^{\left(1\right)}|x_{n}\rangle .$$

Apparently, ${\mathrm{NOT}}^{\left(n\right)}$ always acts on the last bit of any register of $H^{\left(n\right)}$.

The smallest space where the Toffoli-gate can be defined is the space $H^{\left(3\right)}$.

**Definition** **11** (The Toffoli-gate on the space $H^{\left(3\right)}$)**.** *The* Toffoli-gate *on* $H^{\left(3\right)}$ *is the linear operator* $T^{\left(1,1,1\right)}$ *that satisfies the following condition for every element* |x,y,z〉 *of the canonical basis:*

$$T^{\left(1,1,1,\right)}|x,y,z\rangle :=\left\{\begin{array}{c} |x,y,\;x⊓y\rangle ,\;\;if\;\;z=0;\\ |x,y,\;{\left(x⊓y\right)}^{\prime }\rangle ,\;\;if\;\;z=1 \end{array}\right.$$

*(where* ⊓ *and* ′ *are the* infimum *and the* complement *of the two-valued Boolean algebra based on the set* $\left\{0,1\right\}$*).*

Thus, the Toffoli-gate leaves unchanged the first two bits |x〉 and |y〉 (which represent the *control-bits*); while the third bit |z〉 (representing the *target-bit*) is transformed into

- the bit corresponding to the Boolean conjunction of the two control-bits, when z=0;
- the bit corresponding to the Boolean negation of the conjunction of the two control-bits, when z=1.

Like the negation-gate, the Toffoli-gate also can be generalized to higher-dimensional spaces. For any m,n≥1, the Toffoli-gate $T^{\left(m,n,1\right)}$ on the space $H^{\left(m+n+1\right)}$ is defined as follows:

$$T^{\left(m,n,1\right)}|x_{1},\ldots ,x_{m},y_{1},\ldots ,y_{n},z\rangle :=$$

$$|x_{1},\ldots ,x_{m-1},y_{n-1},y_{1},\ldots ,y_{n-2}\rangle ⊗T^{\left(1,1,1\right)}|x_{m},y_{n},z\rangle .$$

The Toffoli-gate has a special logical interest, since it allows us to define a quantum logical conjunction that behaves as a reversible operation. For any choice of two natural numbers m,n (such that m,n≥1) the reversible conjunction ${\mathrm{AND}}^{\left(m,n\right)}$ is dealt with as a holistic monadic operator that acts on global pieces of quantum information represented by quregisters of the space $H^{\left(m+n\right)}$. Accordingly, any quregister of $H^{\left(m+n\right)}$ can be regarded as a holistic description of two possible members of the conjunction ${\mathrm{AND}}^{\left(m,n\right)}$, which live in the space $H^{\left(m\right)}$ and $H^{\left(n\right)}$, respectively.

**Definition** **12** (The conjunction on the space $H^{\left(m+n\right)}$)**.** *For any quregister* |ψ〉 *of $H^{\left(m+n\right)}$,*

$${\mathrm{AND}}^{\left(m,n\right)}|\psi \rangle :=T^{\left(m,n,1\right)}(|\psi \rangle ⊗\left|0\rangle \right)$$

*(where the bit* |0〉 *plays the role of an* ancilla*).*

In the particular case of mixed states $\rho \in D(H^{\left(m+n\right)})$ we will write:

$${}^{D}{\mathrm{AND}}^{\left(m,n\right)}\left(\rho \right)\;\;\;\mathrm{for}\;\;\;{\;}^{D}T^{\left(m,n,1\right)}\left(\rho ⊗P_{0}^{\left(1\right)}\right),$$

where ${}^{D}T^{\left(m,n,1\right)}$ is the unitary quantum operation that corresponds to the unitary operator $T^{\left(m,n,1\right)}$.

As a special case consider a register |x,y〉 of the space $H^{\left(2\right)}$. We obtain:

- ${\mathrm{AND}}^{\left(1,1\right)}|x,y\rangle =T^{\left(1,1,1\right)}|x,y,0\rangle =|1,1,1\rangle$, if x=y=1.
- ${\mathrm{AND}}^{\left(1,1\right)}|x,y\rangle =T^{\left(1,1,1\right)}|x,y,0\rangle =|x,y,0\rangle$, if x=0 or y=0.

Thus, ${\mathrm{AND}}^{\left(1,1\right)}$ represents a “good” quantum generalization of classical conjunction. At the same time, this particular form of quantum conjunction gives rise to a characteristic holistic behavior, which is deeply rooted in the holistic features of the quantum formalism (as shown by the following example).

**Example** **2.** *Consider the quregister represented by the following (entangled) Bell-state:*

$$|\psi \rangle =\frac{1}{\sqrt{2}}|1,1\rangle +\frac{1}{\sqrt{2}}|0,0\rangle .$$

*We have:*

- ${\mathrm{AND}}^{\left(1,1\right)}|\psi \rangle =T^{\left(1,1,1\right)}(|\psi \rangle ⊗\left|0\rangle \right)=\frac{1}{\sqrt{2}}|0,0,0\rangle +\frac{1}{\sqrt{2}}|1,1,1\rangle ;$
- ${}^{D}{\mathrm{AND}}^{\left(1,1\right)}\left(P_{|\psi \rangle }\right)=\;\;{\;}^{D}T^{\left(1,1,1\right)}\left(P_{|\psi \rangle }⊗P_{0}^{\left(1\right)}\right)=P_{\frac{1}{\sqrt{2}}|0,0,0\rangle +\frac{1}{\sqrt{2}}|1,1,1\rangle }.$

*Hence,* ${\mathrm{AND}}^{\left(1,1\right)}|\psi \rangle$ *and* ${}^{D}{\mathrm{AND}}^{\left(1,1\right)}\left(P_{|\psi \rangle }\right)$ *represent a pure state of the space* $H^{\left(3\right)}$ *. At the same time, the two reduced states of* $P_{|\psi \rangle }$ *turn out to be one and the same proper mixture (of the space* $H^{\left(1\right)}$ *):*

$$Red^{\left(1\right)}\left(P_{|\psi \rangle }\right)=Red^{\left(2\right)}\left(P_{|\psi \rangle }\right)=\frac{1}{2}I^{\left(1\right)}.$$

*Consequently, the conjunction over the global state* $P_{|\psi \rangle }$ *cannot be represented as the conjunction of the states of the two separate parts:*

$${\mathrm{AND}}^{\left(1,1\right)}\left(P_{|\psi \rangle }\right)\neq {\mathrm{AND}}^{\left(1,1\right)}\left(Red^{\left(1\right)}\left(P_{|\psi \rangle }\right)⊗Red^{\left(2\right)}\left(P_{|\psi \rangle }\right)\right).$$

*This gives rise to a clear violation of the* compositionality-principle.

As semiclassical gates, the negation-gate and the Toffoli-gate are unable to “create” superpositions from register-inputs. Of course, quantum computer theory cannot help using also “genuine quantum gates” that can transform classical inputs (registers) into genuine superpositions (which are responsible for the characteristic parallel structures of quantum computations). An important example is represented by theHadamard-gate.

**Definition** **13** (The Hadamard-gate on the space $H^{\left(1\right)}$)**.** *The Hadamard-gate on* $H^{\left(1\right)}$ *is the linear operator* ${\sqrt{I}}^{\left(1\right)}$ *that satisfies the following conditions:*

$${\sqrt{I}}^{\left(1\right)}|0\rangle =\frac{1}{\sqrt{2}}|0\rangle +\frac{1}{\sqrt{2}}|1\rangle ;\;\;\;{\sqrt{I}}^{\left(1\right)}|1\rangle =\frac{1}{\sqrt{2}}|0\rangle -\frac{1}{\sqrt{2}}|1\rangle .$$

Thus, the Hadamard-gate transforms both bits into two distinct genuine superpositions that might be either true or false with probability $\frac{1}{2}$.

Like the negation and the Toffoli-gate, the Hadamard-gate also can be generalized to higher-dimensional spaces. For any $H^{\left(n\right)}$ (with n>1), the operator ${\sqrt{I}}^{\left(n\right)}$ is defined for every element $|x_{1},\ldots ,x_{n}\rangle$ of the canonical basis as follows:

$${\sqrt{I}}^{\left(n\right)}|x_{1},\ldots ,x_{n}\rangle :=|x_{1},\ldots ,x_{n-1}\rangle ⊗{\sqrt{I}}^{\left(1\right)}|x_{n}\rangle .$$

Quantum computations are performed by quantum circuits. Mathematically any quantum circuit can be described as a finite sequence of gates, all defined on one and the same quantum computational space $H^{\left(n\right)}$. Since gates may be either unitary operators or unitary quantum operations, we will write:

$$C=\left(G_{1}^{\left(n\right)},\ldots ,G_{t}^{\left(n\right)}\right)\;\;\;\;\mathrm{or}\;\;\;\;C^{D}={(}^{D}G_{1}^{\left(n\right)},\ldots ,{\;}^{D}G_{t}^{\left(n\right)}).$$

**Example** **3.** *An interesting example is represented by the following quantum circuit (also called “Mach–Zehnder circuit”):*

$$C=({\sqrt{I}}^{\left(1\right)},\;{\mathrm{NOT}}^{\left(1\right)},\;{\sqrt{I}}^{\left(1\right)}).$$

**Figure 1:**
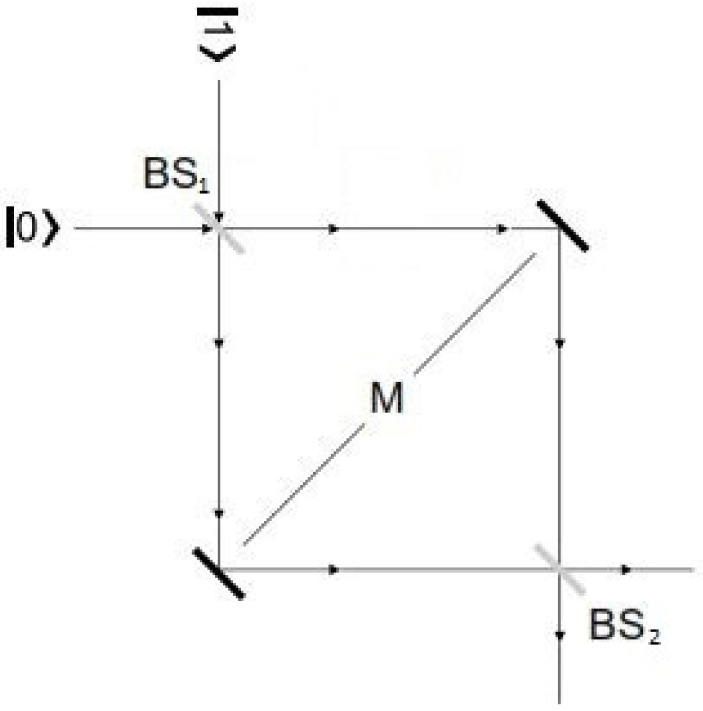
The Mach–Zehnder interferometer.

*As is well known, this circuit can be implemented by a* Mach–Zehnder interferometer *(Figure 1), where the Hadamard-gate is physically realized by a beam-splitter (*BS*), while the negation-gate is realized by a pair of mirrors (*M*).*

*It is worth while recalling how the properties of the Mach–Zehnder circuit and of the Mach–Zehnder interferometers have been an important object of discussion in many foundational debates about quantum theory. The main intuitive “strangeness” is represented by the following mathematical result (which is confirmed by the experimental evidence):*

$${\sqrt{I}}^{\left(1\right)}\;{\mathrm{NOT}}^{\left(1\right)}\;{\sqrt{I}}^{\left(1\right)}|0\rangle =|0\rangle ;\;\;\;{\sqrt{I}}^{\left(1\right)}\;{\mathrm{NOT}}^{\left(1\right)}\;{\sqrt{I}}^{\left(1\right)}|1\rangle =-|1\rangle$$

*(where the bit* |0〉 *is supposed to describe a photon-beam moving along the x-direction, while the bit* |1〉 *describes a photon-beam moving along the y-direction). Such result seems to contradict any classical physical expectation, according to which a beam that has entered into the interferometer along the x-direction, after having crossed the second beam-splitter should be detected with probability-value* $\frac{1}{2}$ *either along the x-direction or along the y-direction.*

## 4. A Logical Abstraction: Quantum Computational Logics

The theory of quantum circuits has inspired a natural logical abstraction, suggesting the development of new forms of quantum logic, that have been called *quantum computational logics*.

As is well known, the prototypical example of quantum logic is the logic created by Birkhoff and von Neumann in their celebrated article “The logic of quantum mechanics”, and usually called Birkhoff and von Neumann’s quantum logic (see [9]). The basic aim of the original quantum logical approach to quantum theory was the development of an abstract analysis of the relationships between the states of a quantum system *S* and the quantum events that may occur to *S*, which can be mathematically represented as projections *P* of the Hilbert space $H_{S}$ associated to *S*. In the semantics of Birkhoff and von Neumann’s quantum logic, the formulas of the quantum logical language are supposed to denote quantum events: projections *P* (of a given space $H_{S}$) to which every state of *S* assigns a well-determined probability-value. At the same time, the basic logical connectives (negation, conjunction, disjunction) are interpreted as special (generally irreversible) algebraic operations that can be defined on the set of all projections *P* of $H_{S}$.

In the logical community, Birkhoff and von Neumann’s quantum logic has been often regarded as a very peculiar and somewhat strange form of non-classical logic, for which some important metalogical questions (like axiomatizability) are still open problems. An axiomatizable version of quantum logic can be obtained by means of a convenient weakening of Birkhoff and von Neumann’s quantum logic. This logic (often called abstract quantum logic) can be semantically characterized by referring to the class of all orthomodular lattices (which contains, as special cases, the orthomodular lattices based on the set of all projections of a Hilbert space, see, for instance, [10]).

The semantics of *quantum computational logics* has been inspired by quite different intuitive ideas, which can be briefly sketched as follows:

- any formula α of a quantum computational language is supposed to denote a piece of quantum information ρ, living in a Hilbert space whose dimension depends on the linguistic complexity of α;
- logical connectives are interpreted as particular gates (for a more detailed exposition see [11,12,13]).

Consequently, unlike the case of traditional quantum logics, the formulas of *quantum computational logics* turn out to have a characteristic dynamic feature, representing possible computation-actions.

In this article we will refer to a minimal (quantum computational) sentential language L, whose alphabet contains:

1. atomic formulas, including two special formulas t and f that denote the *truth* and the *falsity*, respectively;
2. the following logical connectives:
  - the negation ¬, corresponding to the gate negation;
  - the Toffoli-connective ⊺, corresponding to the Toffoli-gate;
  - the Hadamard-connective $\sqrt{id}$, corresponding to the Hadamard-gate.

These connectives simulate, at a syntactical level, the behavior of the corresponding gates. While the negation and the Hadamard-connective are 1-ary connectives (which act on single formulas), the Toffoli-connective is a ternary connective: if α, β are formulas and q is an atomic formula, then ⊺(α,β,q) is a formula. On this basis, recalling the definition of the quantum computational conjunction ${\mathrm{AND}}^{\left(m,n\right)}$, a binary conjunction-connective ∧ can be defined in terms of the Toffoli-connective:

α∧β:=⊺(α,β,f)

(where the false formula f plays the role of a *syntactical ancilla*).

We obtain, in this way, an appropriate logical language for the description of a class of quantum circuits. For instance, the Mach–Zehnder circuit:

$$C=({\sqrt{I}}^{\left(1\right)},\;{\mathrm{NOT}}^{\left(1\right)},\;{\sqrt{I}}^{\left(1\right)})$$

can be naturally described by the following “Mach–Zehnder formula”:

$$\sqrt{id}\;\neg \;\sqrt{id}\;q.$$

A syntactical notion that plays an important semantic role is the concept of atomic complexity of a formula. As we will see, this concept provides a link between the language and the Hilbert-space environment, where the meanings quantum computational formulas are supposed to live.

**Definition** **14** (Atomic complexity)**.** *The atomic complexity* At(α) *of a formula α is the number of occurrences of atomic formulas in α.*

**Example** **4.** *Consider the (contradictory) formula*

α=q∧¬q=⊺(q,¬q,f).

*We have:* At(α)=3.

For any formula α consider the space $H^{\left(At(\alpha )\right)}$ (which is determined by the atomic complexity of α). This space is called *the semantic space* of α, where any piece of quantum information representing a possible *meaning* of α shall live. We will briefly write: $H^{\alpha }$, instead of $H^{\left(At(\alpha )\right)}$.

Any formula α can be decomposed into its parts, giving rise to a syntactical configuration called the *syntactical tree* of α. Let us first consider a particular example.

**Example** **5.** *Consider again the formula*

α=q∧¬q=⊺(q,¬q,f).

*The syntactical tree of α is the following sequence of* levels *, where each level is a particular sequence of subformulas of α:*

$$Level_{3}^{\alpha }=\left(q,q,f\right)$$

$$Level_{2}^{\alpha }=\left(q,\neg q,f\right)$$

$$Level_{1}^{\alpha }=\left(⊺\left(q,\neg q,f\right)\right)$$

In the general case, the levels of the syntactical tree of a given formula α are determined in the following way:

- the bottom level $Level_{1}^{\alpha }$ is (α);
- the top level $Level_{h}^{\alpha }$ is the sequence of atomic formulas occurring in α;
- $Level_{i+1}$ (where 1≤i<h) is obtained by dropping the *principal connective* in all molecular formulas occurring at $Level_{i}$ and by repeating all atomic formulas that occur at $Level_{i}$.

The syntactical tree of any formula α uniquely determines a quantum circuit: a sequence of gates all defined on the semantic space of α. Such sequence is called the *gate tree of α*. For instance, the gate tree of the formula α=⊺(q,¬q,f) is the following circuit:

$$(I^{\left(1\right)}⊗{\mathrm{NOT}}^{\left(1\right)}⊗I^{\left(1\right)},T^{\left(1,1,1\right)}).$$

In fact, the second level of the syntactical tree of α has been obtained from the third level by repeating the first occurrence of q, by negating the second occurrence of q and by repeating f. The first level has been obtained from the second level by applying the Toffoli-connective to the three sentences occurring at the second level.

While the atomic complexity of α corresponds to the width (i.e., the number of wires) of the circuit described by α, the number of levels of the syntactical tree of α (called the height of α) corresponds to the depth (i.e., the number of computational steps) of the circuit in question.

We will now briefly sum up the basic concepts of the *holistic quantum computational semantics*. The notion of holistic model of the language L is based on the weaker notion of holistic map: a map Hol that assigns to each level of the syntactical tree of any formula α a global meaning, represented by a density operator living in the semantic space of α. We have:

$$\mathrm{Hol}:Level_{i}^{\alpha }\;\;\;\mapsto \;\;\;\rho \in D\left(H^{\alpha }\right).$$

On this basis, the meaning that Hol assigns to α is identified with the meaning that Hol assigns to the bottom level of the syntactical tree of α:

$$\mathrm{Hol}\left(\alpha \right)=\mathrm{Hol}\left(Level_{1}^{\alpha }\right).$$

Given a formula α, any holistic map Hol determines the *contextual meaning* with respect to the context Hol(α) of any occurrence of a subformula β in α. Suppose that

$$Level_{i}^{\alpha }=\left(\beta_{i_{1}},\ldots ,\beta_{i_{r}}\right).$$

The *contextual meaning* of $\beta_{i_{j}}$ with respect to the context Hol(α) can be naturally defined using the notion of *reduced state*:

$${\mathrm{Hol}}^{\alpha }\left(\beta_{i_{j}}\right):=Red^{\left(j\right)}\left(\mathrm{Hol}\left(Level_{i}\left(\alpha \right)\right)\right).$$

**Remark** **1.** *Notice how our definition of contextual meaning of a quantum computational formula brings about an interesting connection between the notion of contextuality in linguistic frameworks and the concept of physical contextuality, that plays an important role in many quantum-theoretic problems. As an example, we might recall the debates about the foundational consequences of Kochen and Specker’s theorems and about the logical possibility of deterministic completions of quantum theory via a non-contextual hidden- variable theory.*

The concept of holistic model of the language L can be now defined as a holistic map that satisfies some natural logical restrictions.

**Definition** **15** (Holistic model)**.** *A holistic model of the language* L *is a holistic map* Hol *that satisfies the following conditions:*

*1.*  Hol*preserves the logical form of all formulas. Thus, the meaning of each*$Level_{i}^{\alpha }$*(different from the top level) of the syntactical tree of α is obtained by applying the corresponding gate*$G_{i}^{\alpha }$*(of the gate tree of α) to the meaning of*$Level_{i+1}^{\alpha }$*:*
  $$\mathrm{Hol}\left(Level_{i}^{\alpha }\right)=\;G_{i}^{\alpha }\left(Level_{i+1}^{\alpha }\right).$$
*2.*  Hol*assigns the same contextual meaning to different occurrences (in the syntactical tree of α) of one and the same subformula of α.*
*3.*  *The contextual meanings assigned by*Hol*to the true formula*t*and to the false formula*f*are:*$P_{1}^{\left(1\right)}$*(the truth) and*$P_{0}^{\left(1\right)}$*(the falsity), respectively.*

Suppose that the meaning assigned by a model Hol to a formula α is a pure state, whose form is:

$$c_{1}|\psi_{1}\rangle +\ldots +c_{n}|\psi_{n}\rangle \;\;\;\;\left(\mathrm{where}\;\;\;\;\;c_{i}\neq 0\right).$$

From an intuitive point of view Hol(α) can be regarded as a vague, ambiguous idea that alludes to other ideas (represented by the pieces of information $|\psi_{1}\rangle ,\ldots ,|\psi_{n}\rangle$) that are, in a sense, all *co-existent*. Notice that any meaning Hol(α) represents a kind of autonomous semantic context that is not necessarily correlated with the meanings of other formulas. Generally we have:

$${\mathrm{Hol}}^{\alpha }\left(\gamma \right)\neq {\mathrm{Hol}}^{\beta }\left(\gamma \right).$$

Hence, one and the same formula may receive different contextual meanings in different contexts. As, in fact, happens in the case of our normal use of natural languages.

The following Lemma (which might appear *prima facie* obvious) asserts a highly non-trivial property that plays an important role in the development of the holistic quantum computational semantics (for a proof of Lemma 2 see [13]).

**Lemma** **3.** *Consider a formula γ and let η be a subformula of γ. For any model* Hol *and for any formula β there exists a model* ^*^ Hol *such that:*

$${}^{*}{\mathrm{Hol}}^{\gamma \wedge \beta }\left(\eta \right)={\mathrm{Hol}}^{\gamma }\left(\eta \right).$$

The concepts of truth, validity and logical consequence (in the framework of the holistic quantum computational semantics) can be now defined as follows.

**Definition** **16** (Truth, validity and logical consequence)**.**

*1.*  ${⊧}_{\mathrm{Hol}}\alpha$*(α is* true *in a model* Hol*) iff $p_{1}\left(\mathrm{Hol}\left(\alpha \right)\right)=1$.*
*2.*  ⊧α*(α is* valid*) iff for any model Hol, ${⊧}_{\mathrm{Hol}}\alpha$.*
*3.*  α⊧β*(β is a logical consequence of α) iff for any formula γ such that α and β are subformulas of γ and for any model Hol,*
  $$p_{1}\left({\mathrm{Hol}}^{\gamma }\left(\alpha \right)\right)\leq p_{1}\left({\mathrm{Hol}}^{\gamma }\left(\beta \right)\right).$$

Apparently, both truth and logical consequence are, in this semantics, probabilistic concepts, based on the probability-function $p_{1}$. In spite of the strong contextual features of the holistic quantum computational semantics, one can prove that this holistic notion of *logical consequence* satisfies the transitivity-property.

**Theorem** **4.** α⊧βandβ⊧δ⇒α⊧δ.

**Proof.**  Assume the hypothesis and suppose, by contradiction, that there exists a model Hol and a formula γ, where α and δ occur as subformulas, such that: $p_{1}\left({\mathrm{Hol}}^{\gamma }\left(\alpha \right)\right)\;≰\;p_{1}\left({\mathrm{Hol}}^{\gamma }\left(\delta \right)\right)$. Consider the formula γ∧β. By Lemma 3 there exists a model ${}^{*}\mathrm{Hol}$ such that for any η that is a subformula of γ: ${}^{*}{\mathrm{Hol}}^{\gamma \wedge \beta }\left(\eta \right)={\mathrm{Hol}}^{\gamma }\left(\eta \right)$. Thus, we have: ${}^{*}{\mathrm{Hol}}^{\gamma \wedge \beta }\left(\alpha \right)={\mathrm{Hol}}^{\gamma }\left(\alpha \right)$ and ${}^{*}{\mathrm{Hol}}^{\gamma \wedge \beta }\left(\delta \right)={\mathrm{Hol}}^{\gamma }\left(\delta \right)$. Since we have assumed (by contradiction) that $p_{1}\left({\mathrm{Hol}}^{\gamma }\left(\alpha \right)\right)≰p_{1}\left({\mathrm{Hol}}^{\gamma }\left(\delta \right)\right)$, we obtain: $p_{1}{(}^{*}{\mathrm{Hol}}^{\gamma \wedge \beta }\left(\alpha \right))≰\;p_{1}\left({}^{*}{\mathrm{Hol}}^{\gamma \wedge \beta }\left(\delta \right)\right)$, against the hypothesis (and the transitivity of ≤), which imply: $p_{1}{(}^{*}{\mathrm{Hol}}^{\gamma \wedge \beta }\left(\alpha \right))\leq \;p_{1}\left({}^{*}{\mathrm{Hol}}^{\gamma \wedge \beta }\left(\beta \right)\right)$; $p_{1}\left({}^{*}{\mathrm{Hol}}^{\gamma \wedge \beta }\left(\beta \right)\right)\leq \;p_{1}{(}^{*}{\mathrm{Hol}}^{\gamma \wedge \beta }\left(\delta \right))$; ${}^{*}{\mathrm{Hol}}^{\gamma \wedge \beta }\left(\alpha \right)\leq \;p_{1}\left({}^{*}{\mathrm{Hol}}^{\gamma \wedge \beta }\left(\delta \right)\right)$. ☐

The logic that is semantically characterized by the concept of logical consequence defined in Definition 16 has been called *holistic quantum computational logic* (HQCL). One is dealing with a very *weak* form of logic, where many important *logical arguments* (valid either in classical logic or in Birkhoff and von Neumann’s quantum logic) may be violated.

The following two Theorems sum up some important logical arguments that are either valid or possibly violated in the framework of the logic HQCL (proofs can be found in [13]).

**Theorem** **5.**

*1.*  α⊧α
*2.*  α∧β⊧α;α∧β⊧β
*3.*  α⊧β⟹α∧δ⊧β
*4.*  α⊧¬¬α;¬¬α⊧α
*5.*  α⊧β⟹¬β⊧¬α
*6.*  f⊧β;β⊧t
*7.*  $\sqrt{id}\sqrt{id}\alpha ⊧\alpha ;\alpha ⊧\sqrt{id}\sqrt{id}\alpha$
*8.*  $\sqrt{id}\left(\alpha \wedge \beta \right)⊧\sqrt{id}f;\;\;\;\sqrt{id}f⊧\sqrt{id}\left(\alpha \wedge \beta \right)$

**Theorem** **6.**

*1.*  ⊭¬(α∧¬α)
*2.*  α⊭α∧α
*3.*  α∧β⊭β∧α
*4.*  α∧(β∧γ)⊭(α∧β)∧γ

Thus, conjunctions are generally non-idempotent, non-commutative and non-associative. Such violations can be explained by recalling the contextual behavior of quantum meanings. It may happen that:

$$p_{1}\left({\mathrm{Hol}}^{\delta }\left(\alpha \right)\right)\neq p_{1}\left({\mathrm{Hol}}^{\delta }\left(\alpha \wedge \alpha \right)\right)$$

$$p_{1}\left({\mathrm{Hol}}^{\delta }\left(\alpha \wedge \beta \right)\right)\neq p_{1}\left({\mathrm{Hol}}^{\delta }\left(\beta \wedge \alpha \right)\right)$$

$$p_{1}\left({\mathrm{Hol}}^{\delta }\left(\alpha \wedge \left(\beta \wedge \gamma \right)\right)\right)\neq p_{1}\left({\mathrm{Hol}}^{\delta }\left(\left(\alpha \wedge \beta \right)\wedge \gamma \right)\right).$$

All this seems to be strongly in agreement with a number of informal ways of reasoning where conjunctions are frequently used as non-idempotent, non-commutative and non-associative logical operations. As is well known, the semantics of natural languages is essentially *holistic and contextual*. We need only think how children learn their mother-language, showing an extraordinary capacity of understanding and using correctly the contextual meanings of expressions that occur in different contexts. And it often happens that the meaning of a global expression is grasped and used in a clear and correct way, while the meanings of its parts appear more vague and ambiguous.

Different forms of *holistic quantum computational logics* (also in a first-order version) can be applied to investigate semantic phenomena where holism, contextuality and ambiguity play an important role, as happens not only in the case of natural languages but also in the languages of art (say, poetry or music, for instance, see [2]). Of course the holistic quantum computational semantics does not forbid compositional semantic situations, which can be described as special cases of the holistic semantics. Interestingly enough, conjunctions are always commutative and associative, but generally non- idempotent in the framework of the compositional fragment of the holistic quantum semantics.

## 5. Quantum Epistemic Operations and Quantum Probabilities

Quantum information has brought about some intriguing epistemic situations, that have inspired new ideas in the field of epistemic logics. Is it possible to represent epistemic operators as special examples of operations in a Hilbert space environment? This question admits a positive answer.

A characteristic feature of the quantum computational approach to epistemic logic is the use of the notion of *truth-perspective*: each epistemic agent (say, Alice, Bob, …) is supposed to be associated to a particular truth-perspective that represents his/her idea of truth. Truth-perspective changes may give rise to some interesting relativistic-like epistemic effects: if Alice and Bob have different truth-perspectives, Alice might *see* a kind of deformation in Bob’s logical behavior (see [14]).

From the mathematical point of view we assume that the choice of a given truth-perspective is determined by the choice of a particular orthonormal basis of the Hilbert space $C^{2}$. In Section 2 we have seen how the truth-concept $P_{1}^{\left(n\right)}$ (of $H^{\left(n\right)}$) has been defined by referring to the canonical basis of the space. But, of course, the choice of a particular basis of a given Hilbert space is a matter of convention. Consider the space $C^{2}$. Any orthonormal basis of this space can be described as determined by the application of a unitary operator T to the elements of the canonical basis $\left\{|0\rangle ,|1\rangle \right\}$. We can think that the operator T gives rise to a change of *truth-perspective*. While the classical truth-values *Truth* and *Falsity* have been identified with the two bits |1〉 and |0〉, assuming a different basis corresponds to a different idea of *Truth* and *Falsity*. Since any basis-change in $C^{2}$ is determined by the choice of a particular unitary operator, we can identify a *truth-perspective* with a unitary operator T of $C^{2}$. We will write:

$$|1_{T}\rangle =T|1\rangle ;\;|0_{T}\rangle =T|0\rangle ,$$

and we will assume that $|1_{T}\rangle$ and $|0_{T}\rangle$ represent, respectively, the truth-values *Truth* and *Falsity* of the truth-perspective T. The *canonical truth-perspective* is, of course, determined by the identity operator $I^{\left(1\right)}$. We will indicate by $B_{T}^{\left(1\right)}$ the orthonormal basis determined by T; while $B_{I}^{\left(1\right)}$ will represent the canonical basis. From a physical point of view, we can suppose that each truth-perspective is associated to an apparatus that allows one to measure a given observable.

Any unitary operator T of $H^{\left(1\right)}$ (representing a truth-perspective) can be naturally extended to a unitary operator $T^{\left(n\right)}$ of $H^{\left(n\right)}$ (for any n>1):

$$T^{\left(n\right)}|x_{1},\ldots ,x_{n}\rangle =T|x_{1}\rangle ⊗\ldots ⊗T|x_{n}\rangle .$$

Accordingly, any choice of a unitary operator T of $H^{\left(1\right)}$ determines an orthonormal basis $B_{T}^{\left(n\right)}$ for $H^{\left(n\right)}$ such that:

$$B_{T}^{\left(n\right)}=\left\{T^{\left(n\right)}|x_{1},\ldots ,x_{n}\rangle :|x_{1},\ldots ,x_{n}\rangle \in B_{I}^{\left(n\right)}\right\}.$$

Instead of $T^{\left(n\right)}|x_{1},\ldots ,x_{n}\rangle$ we will also write: $|x_{1_{T}},\ldots ,x_{n_{T}}\rangle$. The elements of $B_{T}^{\left(1\right)}$ will be called the T-*bits* of $H^{\left(1\right)}$; while the elements of $B_{T}^{\left(n\right)}$ will represent the T-*registers* of $H^{\left(n\right)}$.

The notions of *truth*, *falsity* and *truth-probability* can be now generalized to any truth-perspective T.

**Definition** **17** (T-true and T-false registers)**.**

- $|x_{1_{T}},\ldots ,x_{n_{T}}\rangle$*is a*T-true register *iff*$|x_{n_{T}}\rangle =|1_{T}\rangle ;$
- $|x_{1_{T}},\ldots ,x_{n_{T}}\rangle$*is a*T-false register *iff*$|x_{n_{T}}\rangle =|0_{T}\rangle .$

**Definition** **18** (T-truth and T-falsity)**.**

- *The*T-truth *of*$H^{\left(n\right)}$*is the projection operator*${}^{T}P_{1}^{\left(n\right)}$*that projects over the closed subspace spanned by the set of all T- true registers;*
- *the*T-falsity *of*$H^{\left(n\right)}$*is the projection operator*${}^{T}P_{0}^{\left(n\right)}$*that projects over the closed subspace spanned by the set of all T- false registers.*

**Definition** **19** (T-probability)**.** *For any $\rho \in D(H^{\left(n\right)})$,*

$$p_{1}^{T}\left(\rho \right):=\mathrm{tr}\left(\rho \;{\;}^{T}P_{1}^{\left(n\right)}\right).$$

It is worth while noticing that, unlike the probability function $p_{1}$, the relative probability function $p_{\rho }$ cannot be reasonably generalized to different truth-perspectives: as we have seen, the definition of *fidelity* does not depend on the choice of a particular basis of the space.

One can show that all gates can be canonically transposed from the canonical truth-perspective to any truth-perspective T. Hence, any quantum circuit C has a corresponding T-version $C^{T}$ (for any truth-perspective T).

In some previous works we have studied a quantum representation of the epistemic operations “to know”, “to believe”, “to understand”, whose properties depend on the notion of T-truth ${}^{T}P_{1}^{\left(n\right)}$ and on the probability-function $p_{1}^{T}$ (see [14,15]). We will now consider another epistemic operation, which is informally used in a number of interesting quantum situations: the operation “being probabilistically informed”.

Let us first recall the definition of a quantum version of the most important concept of epistemic logics: the concept of knowledge-operation.

**Definition** **20** (Knowledge-operation)**.** *A* knowledge-operation *of a Hilbert space* $H^{\left(n\right)}$ *(with respect to the truth-perspective* T*) is a map*

$$K_{T}^{\left(n\right)}:D\left(H^{\left(n\right)}\right)\mapsto D\left(H^{\left(n\right)}\right).$$

*The following conditions are required:*

*(1)*  $K^{\left(n\right)}$*is associated with an* epistemic domain $EpD(K_{T}^{\left(n\right)})$ *that is a subset of $D(H^{\left(n\right)})$;*
*(2)*  $p_{1}^{T}\left(K_{T}^{\left(n\right)}\rho \right)\leq p_{1}^{T}\left(\rho \right)$ *, for any $\rho \in EpD(K_{T}^{\left(n\right)})$.*

As expected, the intuitive interpretation of $K_{T}^{\left(n\right)}\rho$ is the following: the piece of information ρ is known by a given agent whose truth-perspective is T. The knowledge described by $K_{T}^{\left(n\right)}$ is limited by a given epistemic domain, which is intended to represent the information accessible to our agent, relatively to the space $H^{\left(n\right)}$ (the epistemic domain of $K_{T}^{\left(n\right)}$ should not be confused with the domain of $K_{T}^{\left(n\right)}$, which coincides with the set of all density operators of the space: $K_{T}^{\left(n\right)}\rho$ is defined, even if ρ does not belong to the epistemic domain of $K_{T}^{\left(n\right)}$).Whenever ρ belongs to the epistemic domain of $K_{T}^{\left(n\right)}$, it seems reasonable to assume that the probability-values of ρ and $K_{T}^{\left(n\right)}\rho$ are correlated: the probability of the quantum information asserting that “ρ is known” should always be less than or equal to the probability of ρ. Hence, in particular, we have:

$$p_{1}^{T}\left(K_{T}^{\left(n\right)}\rho \right)=1\;\Rightarrow \;p_{1}^{T}\left(\rho \right)=1.$$

But generally, not the other way around. In other words, pieces of quantum information that are certainly known are certainly true (with respect to the truth-perspective in question). This condition is clearly in agreement with a general principle of standard epistemic logics, according to which “knowledge implies truth, but generally not the other way around”.

A knowledge-operation $K_{T}^{\left(n\right)}$ is called *non-trivial* iff for at least one density operator $\rho \in EpD\left(K_{T}^{\left(n\right)}\right),\;\;\;p_{1}^{T}\left(K_{T}^{\left(n\right)}\rho \right)<p_{1}^{T}\left(\rho \right)$.

Can knowledge-operations be always represented as (reversible) gates? This question has a negative answer, as proved by the following theorem (for a proof of this theorem see [15]).

**Theorem** **7.** *Non-trivial knowledge-operations cannot be generally represented as unitary quantum operations.*

Apparently, the “act of knowing” gives rise to a characteristic reversibility-breaking, which is quite similar to what happens in the case of quantum measurements.

We will now introduce the operation “being probabilistically informed” (indicated by $I_{T}^{\left(n\right)}$), which arises in some interesting situations when an epistemic agent (say, Alice) has a given probabilistic information.

**Example** **6.** *As an example, we can refer to a teleportation-experiment, where, at the initial time, Alice has a probabilistic information, represented by the genuine qubit*

$$|\psi \rangle =c_{0}|0\rangle +c_{1}|1\rangle \;\;\;\;(with\;\;\;\;c_{0},c_{1}\neq 1),$$

*that shall be teleported to the “far” Bob. While* Alice *is* probabilistically informed *about the piece of quantum information* |ψ〉 *(with respect to the canonical truth-perspective), one cannot say that “*Alice certainly knows |ψ〉*”. Since* $P_{|\psi \rangle }$ *is supposed to belong to* Alice’s *epistemic domain, we would obtain:*

$$p_{1}\left(P_{|\psi \rangle }\right)=1$$

*(against the hypothesis that* |ψ〉 *is a genuine qubit).*

The intuitive interpretation of $I_{T}^{\left(n\right)}\rho$ is the following: a given epistemic agent, with truth-perspective T, is probabilistically informed about ρ (in the framework of the space $H^{\left(n\right)}$). As happens in the case of the knowledge-operation $K_{T}^{\left(n\right)}$, the operation $I_{T}^{\left(n\right)}$ is associated to an information-domain $InfD\left(I_{T}^{\left(n\right)}\right)\subseteq D\left(H^{\left(n\right)}\right)$, which represents the set of pieces of information that the agent under consideration is able to valuate probabilistically in the domain $D(H^{\left(n\right)})$. Unlike the case of knowledge-operations (which shall satisfy the strong condition (2) of Definition 20) we admit the following possibility (which occurs, for instance, in the case of the teleportation-example):

- $\rho \in InfD(I_{T}^{\left(n\right)})$
- $p_{1}^{T}\left(I_{T}^{\left(n\right)}\rho \right)=1$
- $p_{1}^{T}\left(\rho \right)<1$

**Definition** **21** (Probabilistic information)**.** *A* probabilistic information-operation *of a Hilbert space* $H^{\left(n\right)}$ *(with respect to the truth-perspective* T*) is a map*

$$I_{T}^{\left(n\right)}:D\left(H^{\left(n\right)}\right)\mapsto D\left(H^{\left(n\right)}\right).$$

*The following conditions are required:*

*(1)*  $I_{T}^{\left(n\right)}$*is associated with an* information-domain $InfD(I_{T}^{\left(n\right)})$ *that is a subset of $D(H^{\left(n\right)})$;*
*(2)*  $\rho \in Inf\left(I_{T}^{\left(n\right)}\right)\;\;\;\;\Rightarrow \;\;\;\;p_{1}^{T}\left(I_{T}^{\left(n\right)}\rho \right)=1$ *.*

It is worth while noticing that the domains $EpD(K_{T}^{\left(n\right)})$ and $InfD(I_{T}^{\left(n\right)})$ are not generally closed under the corresponding operations $K_{T}^{\left(n\right)}$ and $I_{T}^{\left(n\right)}$. Hence, the phenomenon of “epistemic self-consciousness” is here avoided: Alice might know something (or might be probabilistically informed about something) without knowing of knowing it (without being informed of being informed about it).

Every epistemic agent can be naturally associated to an epistemic situation, which is characterized by the choice of a truth-perspective, of a knowledge-operation and of a probabilistic information-operation (in any space $H^{\left(n\right)}$).

**Definition** **22** (Epistemic situation of an agent)**.** *Let* i *represent an epistemic agent. An* epistemic situation *for* i *is a system*

$${\mathrm{EpSit}}_{i}=\;\left(T_{i},\;EpD_{i},\;\;\;InfD_{i},\;\;\;K_{i},\;\;\;I_{i}\right),$$

*where:*

*(1)*  $T_{i}$*represents the truth-perspective of*i*.*
*(2)*  $EpD_{i}$*is a map that assigns to any*n≥1*a set*$EpD_{i}^{\left(n\right)}\subseteq D\left(H^{\left(n\right)}\right)$*that represents the information accessible to*i*in the information-environment $D(H^{\left(n\right)})$.*
*(3)*  $InfD_{i}$*is a map that assigns to any*n≥1*a set*$InfD_{i}^{\left(n\right)}\subseteq EpD_{i}^{\left(n\right)}$*that represents the information that*i*is able to valuate probabilistically in the information-environment $D(H^{\left(n\right)})$.*
*(4)*  $K_{i}$*is a map that assigns to any*n≥1*a knowledge-operation*$K_{T_{i}}^{\left(n\right)}$*(defined on*$H^{\left(n\right)}$*), which describes the knowledge of*i*with respect to the information-environment*$D(H^{\left(n\right)})$*. The epistemic domain associated to the operation*$K_{T_{i}}^{\left(n\right)}$*is the set $EpD_{i}^{\left(n\right)}$.*
*(5)*  $I_{i}$*is a map that assigns to any*n≥1*an information-operation*$I_{T_{i}}^{\left(n\right)}$*(defined on*$H^{\left(n\right)}$*), which describes the probabilistic information of*i*with respect to the information-environment*$D(H^{\left(n\right)})$*. The information-domain associated to the operation*$I_{T_{i}}^{\left(n\right)}$*is the set $InfD_{i}^{\left(n\right)}$.*
*(6)*  $\forall \rho \in D\left(H^{\left(n\right)}\right):p_{1}^{T_{i}}\left(K_{T_{i}}^{\left(n\right)}\rho \right)\;\;\leq \;\;p_{1}^{T_{i}}\left(I_{T_{i}}^{\left(n\right)}\rho \right)$*.*
  The probability of knowing a given information is less than or equal to the probability of having a probabilistic valuation about it. But, generally, not the other way around.

On this basis one can develop a *quantum epistemic semantics* for a first-order language that can be express formulas like:

- Kaα (*a* knows α);
- Iaα (*a* is probabilistically informed about α).

This semantics allows us to represent and to justify a number of significant features of our normal use of epistemic notions in the framework of natural language. Interestingly enough, the unpleasant phenomenon of logical omniscience is here avoided. Due to the limits of epistemic domains, Alice might know a given sentence without knowing all its logical consequences. Furthermore, knowledge and probabilistic information are not generally closed under logical conjunction, in accordance with what happens in the case of concrete memories both of human and of artificial intelligence (see [14]).

In conclusion, we have seen how the holistic quantum computational semantics provides some useful abstract tools that can be naturally applied to a formal analysis of concepts and problems in fields that may be far apart from microphysics. Some interesting examples concern the use of crucial epistemic concepts (like knowledge, information, belief, understanding) both in the case of rigorous scientific arguments and in some informal ways of reasoning. Other examples regard the role of ambiguity, vagueness, uncertainty and contextuality either in scientific theories or in our normal use of natural languages or in the languages of art (say, poetry or music).

According to some traditional philosophical views, ambiguity and holism represent characteristic features of human thought that cannot be adequately analyzed in the framework of scientific theories, whose semantics is supposed to be essentially “sharp” and “analytical”. Interestingly enough, quantum logics (in their different versions) have provided a significant bridge that might fill a gap between humanistic and scientific disciplines.
